# File-based localization of numerical perturbations in data analysis pipelines

**DOI:** 10.1093/gigascience/giaa106

**Published:** 2020-12-02

**Authors:** Ali Salari, Gregory Kiar, Lindsay Lewis, Alan C Evans, Tristan Glatard

**Affiliations:** Department of Computer Science and Software Engineering, Concordia University, Montreal, QC, Canada; Department of Biomedical Engineering, McGill University, Montreal, QC, Canada; Montreal Neurological Institute, McGill University, Montreal, QC, Canada; Department of Biomedical Engineering, McGill University, Montreal, QC, Canada; Department of Biomedical Engineering, McGill University, Montreal, QC, Canada; Montreal Neurological Institute, McGill University, Montreal, QC, Canada; Department of Computer Science and Software Engineering, Concordia University, Montreal, QC, Canada

**Keywords:** Reproducibility, Operating Systems, Neuroimaging, Pipelines

## Abstract

**Background:**

Data analysis pipelines are known to be affected by computational conditions, presumably owing to the creation and propagation of numerical errors. While this process could play a major role in the current reproducibility crisis, the precise causes of such instabilities and the path along which they propagate in pipelines are unclear.

**Method:**

We present Spot, a tool to identify which processes in a pipeline create numerical differences when executed in different computational conditions. Spot leverages system-call interception through ReproZip to reconstruct and compare provenance graphs without pipeline instrumentation.

**Results:**

By applying Spot to the structural pre-processing pipelines of the Human Connectome Project, we found that linear and non-linear registration are the cause of most numerical instabilities in these pipelines, which confirms previous findings.

## Introduction

Numerical perturbations resulting from variations in computational environments affect data analyses in various fields, but identifying the origin of these perturbations in complex pipelines remains challenging. In some cases, small perturbations resulting from changes in operating system (OS) versions [1], hardware [2], or parallelization parameters [3] result in substantially different analysis outcomes, owing to the propagation and amplification of floating-point errors. While the existence of such numerical errors is well known [4], their impact on scientific computations has multiplied with the rise of the Big Data era, owing to the sustained growth of datasets, the increasing complexity of analysis pipelines, and the diversification of computing infrastructures. To better understand and correct these effects, efficient tools are needed to assist pipeline developers in the comparison of results obtained across different conditions.

In neuroimaging, our primary application field, data analyses often consist of hundreds of computational processes— often coming from multiple toolboxes—that are aggregated to perform a specific function. For instance, the fMRIprep pipeline [5] assembles software blocks from FSL [6], AFNI [7], FreeSurfer [8], and ANTS [9] to provide a state-of-the art functional magnetic resonance imaging (fMRI) processing tool with minimal user input. Another example are the pipelines of the Human Connectome Project (HCP) [10] that combine tools from FSL and FreeSurfer to pre-process structural, functional, and diffusion data from their uniquely high-fidelity open dataset. In both cases, pipelines leverage toolboxes that are widely trusted in the community, yet, at the same time substantial variations in results have been observed in these toolboxes resulting from minor data or infrastructure perturbations [1, 11–13], suggesting that further investigation of their numerical conditioning is required. For such complex pipelines, a lightweight solution has to be found to perform such evaluations with limited code instrumentation.

Numerical evaluations are traditionally performed using techniques such as interval arithmetics [14] that require complete code rewrites and are therefore barely applicable to complex pipelines. Recently, Monte Carlo arithmetic (MCA) [15,16] provided a practical way to evaluate the uncertainty of numerical results without the need to rewrite the application in a different paradigm. By perturbing floating-point computations, it introduces a controllable amount of noise in the pipelines, effectively sampling results from a random distribution. While this technique is appealing, it is hindered by 2 main issues that make it impractical at the scale of a complete pipeline. First, it requires all software components to be recompiled for MCA instrumentation, which is not always feasible. Second, it multiplies the execution time by a factor of 10–100, which is impractical when executions already take a few hours to complete.

We present Spot, a tool to identify the source of numerical differences in complex pipelines without instrumentation. Using system-call interception through the ReproZip tool [17], Spot traverses graphs of processes and intermediary files to pinpoint the pipeline components that are unstable across execution conditions. When differences start accumulating, effectively masking any further instability, it restores clean data copies through a set of wrapper scripts. Wrapper scripts are also used to restore temporary data that might have been deleted during the execution and to disambiguate files that have been written by multiple processes. The remainder of this article presents the design of Spot and its application to pre-processing pipelines of the HCP.

## Tool Description

**Figure 1:**
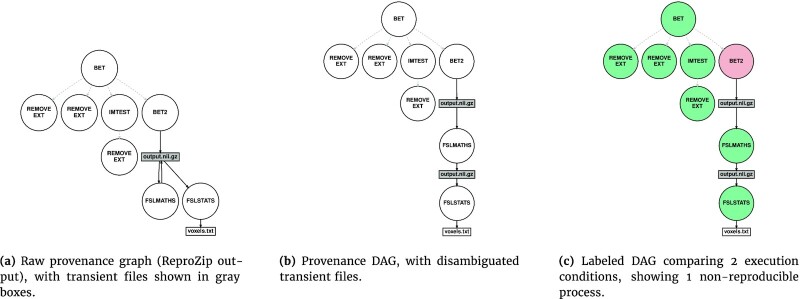
Provenance graphs created from the example pipeline in Listing 1. Processes are represented with circles, files with rectangles, and read/write accesses with plain edges. For convenience, the process tree is also shown, with gray dashed edges. Processes forked by bet were captured by ReproZip although they did not appear in Listing 1. Processes associated with executables located in /usr/bin/ or /bin/ are not shown. (a) Raw provenance graph (ReproZip output), with transient files shown in gray boxes. (b) Provenance DAG, with disambiguated transient files. (c) Labeled DAG comparing 2 execution conditions, showing 1 non-reproducible process. Green indicates reproducible, and pink, non-reproducible processes.

Spot identifies the components in a pipeline, at the resolution level of a system process, that produce different results in different execution conditions. First, a directed bipartite provenance graph is recorded for each pipeline execution, where nodes represent application processes and files, and edges represent read and write file accesses (Fig. 1a). Second, transient files, i.e., files that are either deleted during pipeline execution or modified by multiple processes, are identified and disambiguated, resulting in a provenance directed acyclic graph (DAG) in which file nodes have a single parent (in-degree of 1) (Fig. 1b). DAGs produced in different conditions are then compared, in a step-by-step execution that prevents the propagation of differences in the pipeline (Fig. 1c). The resulting labeled graph identifies the non-reproducible processes in the pipeline.

To ensure that a file can be unambiguously associated with the process that created it, we assume that the pipeline can be transformed such that:

- Processes do not run concurrently;
- Each process sequentially reads, computes, and writes.

In practice, pipeline processes may still run concurrently provided that they do not write concurrently to the same files. A process may also interleave file writes with computing, e.g., when different file blocks are processed sequentially. However, only a single version of the file must eventually be made available to the other processes. In particular, in case a process deletes a file that it had created itself, this file must not be used by any other process. Finally, we also require processes to be associated to a command line (executable and arguments) to facilitate process instrumentation.

**Listing 1:**
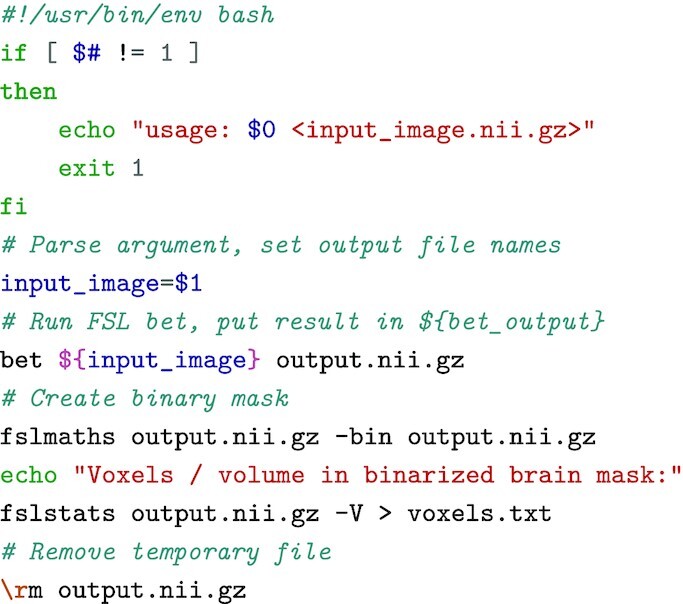
Example pipeline that computes the volume of the brain from a T1 image.

### Recording provenance graphs

We use ReproZip  [17] to capture (i) the set of processes created by the pipeline and (ii) the set of files read and written by each process, including temporary files. ReproZip collects this information through the ptrace() system call, with no required instrumentation of the pipeline. Using the ReproZip trace, Spot reconstructs a provenance graph by creating process and file nodes and by adding directed edges corresponding to file reads and writes (Fig. 1a). We assume that provenance graphs are identical for the ReproZip traces obtained from the same subjects in different OSs.

Provenance graphs are often data dependent, owing to variations in input data that may trigger differing branching or looping patterns across executions, for example. Some of these differences can be neglected: e.g., when a data decompression step is present at the beginning of the execution for some subjects only. Other differences cannot: e.g., when entirely different processing paths are used for different datasets. Spot includes helpers to identify different instances of provenance graphs, such as supporting the clustering of process trees, where nodes are processes and edges are fork() or clone() system calls, using the tree edit distance [18] implemented in Python’s zss package.

### Capturing transient files

We capture temporary files by replacing every process *P* by a wrapper that first calls *P* and then saves the produced temporary files to a read-only directory. This process replacement is done by pre-pending to the PATH environment variable a directory that contains a wrapper script named after the executable called by *P*.

Files written by multiple processes are disambiguated using a similar technique. For a file *F* written by the processes in **P** = {*P*_1_, ..., *P_n_*}, we first check that processes in **P** do not write concurrently to *F*, which would violate our assumptions. Then, we replace every process *P_i_* by a PATH-based wrapper that first calls *P_i_* and then saves *F* to a read-only directory. In this way, successive versions of *F* are preserved for comparison. We finally update the provenance graph accordingly, so that all files in the graph have an in-degree of 1 (Fig. 1b). This operation also makes the provenance graph acyclic because we assumed that a process could only release a single version of a file.

### Labeling processes

After capturing transient files in the first condition (i.e., OS, library version, and so forth), we rerun the pipeline step by step in the second one to label processes. The output files created by a process in both conditions are compared: if no differences are found, the process is marked as reproducible; otherwise, the process is marked as non-reproducible, and the output files produced in the first condition are copied to the second one, to ensure that differences do not propagate further in the pipeline. Processes are instrumented transparently through a modification of the PATH variable similar to the one described previously. By default, differences in output files are identified by comparing file checksums. Other comparison functions can also be defined for specific file types, e.g., to ignore file headers or file sections containing timestamps. Spot finally creates a labeled provenance graph highlighting non-reproducible processes.

Fig. 1c illustrates a hypothetical incremental labeling of the example in Listing 1. Process bet2 is labeled as non-reproducible (pink) because it produces files with differences. To prevent the propagation of these differences, the files produced by bet2 in Condition 2 are replaced with the files produced by bet2 in Condition 1. Processes fslmaths and fslstats are then executed and labeled as reproducible (green) because they produce files without differences.

The labeled graph can differ depending on the order of executions in which condition we capture transient files or execute the pipeline to pinpoint the propagation of differences. Therefore, we run the comparison in both condition orders, and we label a process as non-reproducible (pink) if it creates different results in ≥1 condition order.

### Implementation

Spot is implemented in Python (version ≥3.6). In this work we used Spot version 0.2 and the following version of the Python package dependencies: NumPy v1.19.0 [19] and Pandas v1.0.5 [20] for data manipulations, SciPy v1.5.1 [21] and Scikit-learn v.0.23.1 [22] for the clustering of provenance graphs, Zss v1.2.0 [18] for tree distances, ReproZip v1.0.11 for the capture of provenance traces, Docker v17.05 [23] for the edition of container images, and Boutiques v0.5.25 [24] for uniform pipeline executions.

Software users will mostly have to interact with the Boutiques and ReproZip packages. Boutiques is a flexible description framework for containerized pipelines, required by the pipelines analyzed in Spot. It provides a JSON schema to describe inputs, outputs, and their dependencies. Examples, tutorials, and use documentation are available online [25]. ReproZip intercepts system calls to identify the files and processes involved in a pipeline execution. Before using Spot, users have to collect ReproZip traces of their pipeline executions. Examples in the Spot documentation include ReproZip provenance capture. More documentation on ReproZip is available [26].

## Experiments

We applied Spot to the minimal pre-processing pipelines released by the HCP, a leading initiative in neuroimaging.

### HCP pipelines and dataset

The HCP developed a set of pre-processing pipelines to process structural, functional, and diffusion MRI data acquired in the project. We focus on HCP pre-processing pipelines for structural data, and particularly on PreFreeSurfer and FreeSurfer. A detailed description of the analyses done by these pipelines is available [10]. In summary, the PreFreeSurfer pipeline consists of the following steps:

- Gradient Distortion Correction (DC),
- Alignment and Anatomical Average (AAve), T1w(s), T2w(s),
- Anterior/Posterior Commissure Alignment (ACPC-A),
- Brain Extraction (BExt),
- Bias Field Correction (BFC),
- Atlas Registration (AR).

And the FreeSurfer pipeline consists of the following:

- Image downsampling,
- T1w image registration,
- T1w image segmentation,
- Surface placement,
- Surface registration.

We randomly selected 20 unprocessed subjects from the HCP data release S500 available in the ConnectomDB repository as a subset of the 1200 Subject Release (see Supplementary Table S1). For each subject, available data consisted of 1 or 2 T1-weighted images and 1 or 2 T2-weighted images, with 256 × 320 × 320 voxels of size 0.7 × 0.7 × 0.7 mm. Acquisition protocols and parameters are detailed in [27].

### Data processing

We built Docker images for the HCP pre-processing pipelines v3.19.0 (PreFreeSurfer and FreeSurfer) in CentOS6.9 (Final) and CentOS7.4 (Core), available on DockerHub. Container images contain the HCP software dependencies, including FSL (version 5.0.6), FreeSurfer (version 5.3.0-HCP, CentOS4 build), and Connectome Workbench (version 1.0).

We processed the 20 subjects with PreFreeSurfer and FreeSurfer, using the 2 CentOS versions. The PreFreesurfer results obtained in CentOS6 were used as the input of FreeSurfer in both conditions. We also used the ReproZip trace file captured in CentOS6 for labeling the processes in both pipelines. Each subject was processed twice on the same OS to detect within-OS variability coming from pseudo-random operations. We compared pipeline results using FreeSurfer tools mri_diff, mris_diff, and lta_diff, to ignore execution-specific information such as file path or timestamps. To compare segmentations *X* and *Y*, we used the Dice coefficient defined as follows:

\begin{eqnarray*}
\mathrm{Dice}=\frac{2|X \cap Y|}{|X| + |Y|}. \end{eqnarray*}

The Dice coefficient [28] is a commonly used metric to validate medical image segmentation. Dice values range from 0 to 1, with 1 indicating a perfect overlap between 2 segmentation results and 0 indicating no overlap. Alternatively, the Jaccard coefficient [29] could be used; there is a direct correspondence between both metrics.

## Results

All experiments were run on a machine with a 3.4 GHz, 8-core Intel Core i7 processor, 32 GB of RAM, CentOS 7.3.1611, and Linux kernel version 3.10. The processing time, output file size, number of file accesses, and number of processes observed in PreFreeSurfer and FreeSurfer are reported in Table 1. The scripts and analyses used to create the figures in this section are available at GitHub [30].

**Table 1.** Execution statistics of the pipelines per subject

Statistic	PreFreeSurfer, mean (SE)	FreeSurfer, mean (SE)
Processing time (minutes)	106.67 (2.68)	650.25 (8.88)
Output file size (GB)	2.8 (0.10)	4.15 (0.15)
No. of file accesses	94,089 (2,645)	62,729 (984)
No. of processes	8,731 (198)	4,031 (47)

### Within-OS differences

We did not observe any within-OS difference in PreFreeSurfer. In FreeSurfer, we identified 2 processes leading to within-OS differences due to the use of pseudo-random numbers: image registration with mri_segreg, and cortical surface curvature estimations with mris_curvature. Fixing the random seed used in FreeSurfer removed these differences.

### Between-OS differences in PreFreeSurfer

We identified 4 types of subjects with different PreFreeSurfer provenance graphs (Table 2). Differences between subject types came from different numbers of T1 and T2 images in the raw data. We verified that the provenance graphs were identical for all subjects of the same type, for both versions of CentOS.

**Table 2.** Types of provenance graphs in PreFreeSurfer

Type	No. of subjects	No. of images	
		T1w	T2w
1	9	2	2
2	8	1	1
3	1	1	2
4	2	2	1

**Figure 2:**
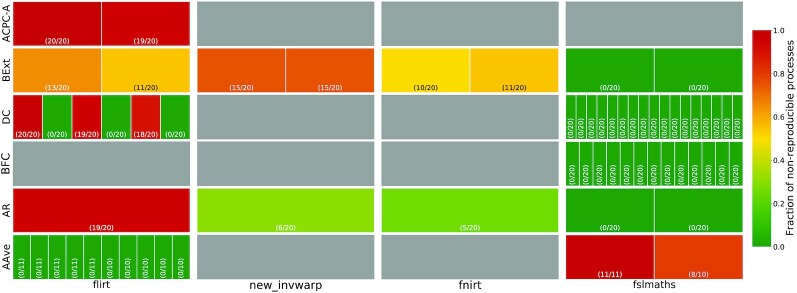
Heat map of non-reproducible processes across PreFreeSurfer pipeline steps. Each cell represents the occurrence of a particular command line in a pipeline step among Anatomical Average (AAve), Anterior/Posterior Commissure Alignment (ACPC-A), Brain Extraction (BExt), Bias Field Correction (BFC), or Atlas Registration (AR). Cell labels indicate the fraction of subjects for which the corresponding process was not reproducible. For example, the flirt tool was invoked 6 times in step DC for each of the 20 subjects: 1 instance was not reproducible in all subjects, 3 instances were always reproducible, 1 instance was not reproducible in 18 subjects, and 1 instance was not reproducible in 19 subjects. Gray cells indicate that the process did not occur in the corresponding pipeline step.

**Figure 3:**
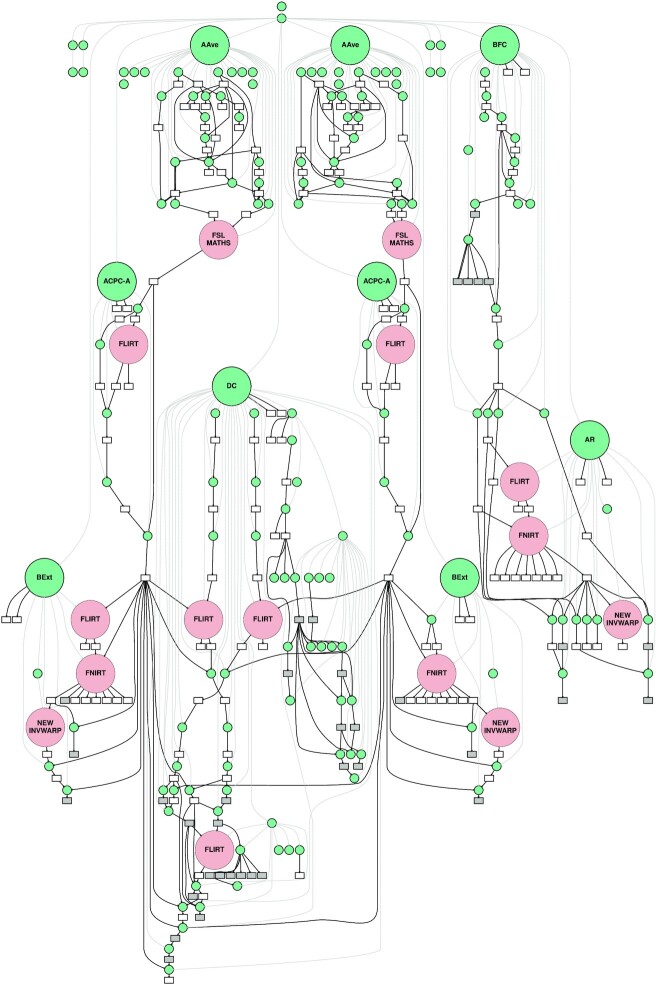
A complete provenance graph from the PreFreesurfer pipeline. Node labels use the same abbreviations as in Fig. 2. For better visualization, processes associated with commands in /bin or /usr/bin were omitted, as well as imtest, imcp, remove_ext, fslval, avscale, and fslhd.

Fig. 2 shows the frequency of non-reproducible pipeline processes in PreFreeSurfer. The processes identified as non-reproducible were observed in linear registration with FSL flirt (in ACPC-A, BExt, DC, and AR), in non-linear registration with FSL fnirt (in BExt and AR), and in image warping with FSL new_invwarp (in BExt and AR). Differences were also observed in image mean computations with FSL maths (in AAve). Fig. 3 shows a complete PreFreeSurfer labeled DAG, localizing the observed differences in the entire pipeline, for a given subject.

**Figure 4:**
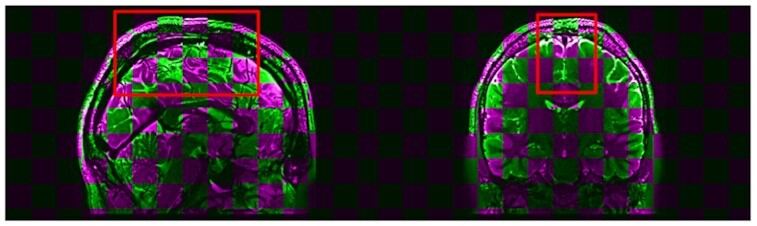
Differences between T2 fnirt results in PreFreeSurfer’s Brain Extraction (CentOS6 vs CentOS7). The colored squares indicate results obtained with CentOS6 (purple) and CentOS7 (green). The red boxes highlight regions with significant differences between the 2 OSs. An animated version of the comparison is available herefor better visualization.

Fig. 4 compares fnirt results in BExt for a particular subject using the checkerboard pattern, a common method to illustrate the magnitude of the differences in registration results. Differences appear to be visually important, in particular in the areas framed in red, to the point that most experimenters would likely reject such a registration following visual quality control.

### Between-OS differences in FreeSurfer

The only non-reproducible process identified by Spot in FreeSurfer was mris_make_surfaces (cortical and white matter surfaces generation), a dynamically linked executable that produced different results for 10 of 20 subjects.

**Figure 5:**
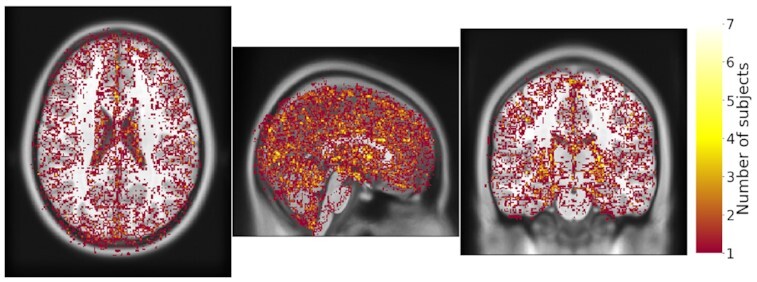
Sum of binarized differences between whole-brain FreeSurfer segmentations obtained from PreFreeSurfer processings in CentOS6 vs CentOS7 (N = 20). Segmentations were resampled and overlaid to the MNI152 volume template. Each voxel shows the number of subjects for which different results were observed between CentOS6 and CentOS7. An animated comparison of segmentations obtained for a particular subject is available here for better visualization.

**Figure 6:**
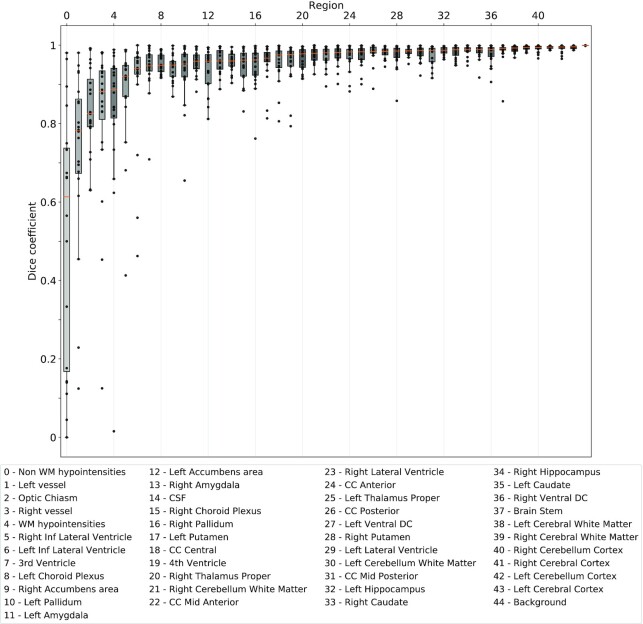
Dice coefficients between regions segmented by FreeSurfer in CentOS6 vs CentOS7 (N = 20), ordered by increasing median values. Each point represents the Dice coefficient between segmentations of a particular region obtained in CentOS6 vs CentOS7 for a given subject. Box brightness is proportional to the logarithm of the corresponding brain region size. CSF: cerebro-spinal fluid, WM: white matter, CC: corpus callosum, DC: Diencephalon.

However, FreeSurfer results still differ between conditions, owing to the propagation of differences created in PreFreeSurfer. We observed the effect of this propagation in FreeSurfer results, as shown in Fig. 5 for whole-brain segmentations. The Dice coefficients associated with the 44 regions segmented by FreeSurfer are shown in Fig. 6, showing that Dice coefficients <0.9 are observed in most regions, and particularly in the smallest ones. However, no significant correlation between the Dice values and the region sizes was found (Pearson coefficient = 0.12, *P* = 0.43).

## Discussion

Our results provide insights on the reproducibility of neuroimaging pipelines, and on the relevance of the approach implemented in Spot for reproducibility studies.

### Key findings

Linear and non-linear registration with FSL were found to frequently lead to differences between results obtained with different OSs. This does not come as a surprise given the instabilities associated with these processes. It also corroborates our previous findings [1], where fMRI pre-processing with FSL was found to vary across OSs starting from the motion correction step, a step that uses FSL’s flirt tool internally. It would be relevant to investigate whether the observed instability of registration processes generalizes to other toolkits or remains specific to FSL. In view of the effect of small data perturbations in a variety of toolboxes and processes, such as cortical surface extraction using FreeSurfer and CIVET [12] or connectome estimation using Dipy [31], it is probable that this observation generalizes widely across toolboxes and requires a deeper investigation of the stability of linear and non-linear registration.

While only a handful of processes were found non-reproducible across the tested OSs, the effects of such instabilities were found to propagate widely in the pipelines and to substantially affect the segmentations created by FreeSurfer. This illustrates the need to conduct reproducibility studies on entire pipelines rather than isolated processes. It also highlights the need for a deeper stability analysis of pipeline processes.

As shown in Fig. 2, the reproducibility of a given tool may vary across subjects and across processing parameters. For instance, linear registration with flirt seems to be fully reproducible in the AAve sub-pipeline, while it is highly non-reproducible in ACPC-A. In BExt, the same tool was found reproducible for some subjects only. Therefore, reproducibility studies need to be performed on several subjects. While this is common practice to some extent in neuroimaging, software tests are often executed only on a single dataset to reduce the associated computational load. Our results show that pipeline tests should encompass enough subjects to cover execution paths adequately.

Our results illustrate the type of variability that can be introduced in neuroimaging results due to OS updates. The numerical noise introduced by OS updates is realistic because such updates are likely to occur throughout the time span of a neuroscience study, but it is also uncontrolled, as it originates in updates of low-level libraries by third-party developers. A possible method to study this problem more comprehensively would be to introduce controlled numerical perturbations in pipelines, which could be done by introducing noise either in the data, or in floating-point computations through MCA [15]. Beauzamy [31] discusses and compares these 2 techniques.

### Spot evaluation

The processes identified by Spot as non-reproducible were all associated with dynamically linked executables. This makes complete sense because statically linked executables are not affected by library updates. Moreover, the hypothetical effects of hardware or Linux kernel updates were not measured because the different OSs were deployed in Docker containers on the same host, i.e., using the same kernel and hardware.

To evaluate the reproducibility of a pipeline, Spot needs to execute it 5 times in order to (1) record a first ReproZip trace, (2) save transient files in the first condition, (3) compare results in the second condition, and repeat Steps 2 and 3 for the other order of execution. It might be possible to further reduce this overhead by executing at Step 2 only the processes depending on transient files, and capturing the transient files for the second condition simultaneously at Step 3.

The target users of the Spot tool are primarily pipeline developers and users who have technical skills for creating Docker containers and Boutiques JSON files. We demonstrated the applicability of our approach by evaluating 2 of the arguably most complex pipelines in neuroimaging. Technically, these pipelines consist of a mix of tools assembled from different toolboxes through a variety of scripts written in different languages. Our file-based approach, notably enabled by ReproZip, was able to analyze these pipelines without requiring their instrumentation, which saved a very substantial technical effort. The assumptions made on the pipeline structure, related to the absence of concurrent writes, were not violated in our analysis and are likely not to impede Spot’s applicability to the most common neuroimaging pipelines.

Spot only tests pipeline reproducibility in the scope of a particular dataset. However, it is very plausible for pipeline processes to exhibit different reproducibility behaviors when executed on different datasets. Therefore, only the lack of reproducibility of a pipeline process could be guaranteed from an analysis with Spot because proving reproducibility would require testing the pipeline on all possible datasets, in all possible environments, which is not feasible. Two elements could be considered in future work to address this issue. First, similar to conventional software testing, a code coverage metric could be developed to assess the fraction of the pipeline code involved in the tested dataset and parameters. This would quantify the representativity of the dataset and pipeline parameters used in the evaluation. Second, statistical risk models could be used to estimate the probability for a process to be reproducible, given a set of observations with no numerical differences. For instance, models described in Botvinik-Nezer et al. [32] could be leveraged for this purpose.

File-based analyses also have limitations related to the granularity at which they operate. Indeed, differences can only be identified at the level of an entire OS process, which can correspond to arbitrary amounts of code. Narrowing down the analysis to particular libraries, functions, or even code sections would require another approach. Similarly, Spot would not be able to detect differences in data not saved in files but instead passed to subsequent processes in memory. A common scenario in neuroimaging pipelines is that tools return results in their standard output, which is parsed by the calling process and passed to subsequent ones through variables.

Computational environments are only one of many factors contributing to the ongoing reproducibility crisis. In fact, sample size selection, publication bias, or methodological flexibility in the analysis are likely to have a stronger effect than numerical perturbations, although to our knowledge no evidence of this is available. We refer to published studies [13, 33–35] for deeper analyses of the associated effects on neuroimaging analyses. It should also be noted that the effects of computational environments and these other factors manifest at different levels: referring to the terminology used by Salari et al. [36], computational environments are associated with "reproducibility," the minimal standard by which identical results should be obtainable from identical data and parameters, while the other aforementioned factors belong to “replicability," the ultimate standard by which independent experimenters should be able to draw similar conclusions from similar experiments. In practice, variability resulting from computational environments manifests during software testing (test results depend on execution platform), deployment on high-performance computing systems (results obtained on local vs high-performance computing systems differ), or software version updates (results obtained before vs after the update differ), while factors related to replicability affect the community more broadly. Ultimately, both reproducibility and replicability should be understood and improved.

## Conclusion

We present Spot, a tool to detect the source of numerical differences in complex pipelines executed in different computational conditions. Spot leverages system-call interception through the ReproZip tool and therefore can be applied to the most complex pipelines without requiring their instrumentation. It is available at the project home page under MIT license.

By applying Spot to the pre-processing pipelines of the HCP, compared in different OSs, we showed that between-OS differences are mostly originating in linear and non-linear image registration tools. Moreover, differences introduced during image registration propagate widely in the pipelines, leading to important variability in whole-brain segmentations.

Future work will investigate in more detail the numerical stability of registration algorithms. Additionally, we plan on using MCA to inject controlled amounts of noise in pipelines and monitor uncertainty propagation and amplification in their results.

## Availability of Source Code and Requirements

- Project name: Spot
- Project home page: https://github.com/big-data-lab-team/spot
- Operating system: Linux
- Programming language: Python (3.6 or higher)
- Main dependencies: ReproZip, Docker, and Boutiques
- Other dependencies: see setup.py
- License: MIT License
- Biotools identifier: spottool
- RRID:SCR_018915
- doi:10.5281/zenodo.3873219

## Data Availability

Snapshots of our code and other supporting data are openly available in the *GigaScience* GigaDB repository [37] and Zenodo [38].

## Additional Files

Supplementary Table S1. Summary of the subjects who participated in the experiments.

## Abbreviations

ACPC-A: Anterior/Posterior Commissure Alignment; AFNI: Analysis of Functional NeuroImages; ANTS: Advanced Normalization Tools; AR: Atlas Registration; AAve: Anatomical Average; BExt: Brain Extraction; BFC: Bias Field Correction; DAG: directed acyclic graph; DC: Distortion Correction; fMRI: functional magnetic resonance imaging; FSL: FMRIB Software Library; HCP: Human Connectome Project; JSON: JavaScript Object Notation; MCA: Monte Carlo arithmetic; NIH: National Institutes of Health; OS: operating system; RAM: random access memory.

## Competing Interests

The authors declare that they have no competing interests.

## Authors' Contributions

Conceptualization: AS, TG; Supervision: TG, ACE; Project Administration: TG, ACE; Investigateion: AS; Formal Analysis: AS, TG; Software: AS; Methodology: AS, TG; Validation: AS, TG; Data: LBL; Funding Acquisition: TG; Writing - Original Draft Preparation: AS, TG; Writing - Review & Editing: GK, LBL.

## Acknowledgments

We thank the reviewers for their thorough and insightul review, which greatly contributed to the improvement of the manuscript. We warmly thank Compute Canada (http://www.computecanada.ca) and Calcul Québec (http://www.calculquebec.ca) for providing the infrastructure used in our experiments. Data and pipelines were provided by the Human Connectome Project, WU-Minn Consortium (Principal Investigators: David Van Essen and Kamil Ugurbil; 1U54MH091657) funded by the 16 NIH Institutes and Centers that support the NIH Blueprint for Neuroscience Research; and by the McDonnell Center for Systems Neuroscience at Washington University.

## Supplementary Material

giaa106_GIGA-D-20-00177_Original_Submission

giaa106_GIGA-D-20-00177_Revision_1

giaa106_GIGA-D-20-00177_Revision_2

giaa106_GIGA-D-20-00177_Revision_3

giaa106_Response_to_Reviewer_Comments_Original_Submission

giaa106_Response_to_Reviewer_Comments_Revision_1

giaa106_Response_to_Reviewer_Comments_Revision_2

giaa106_Reviewer_1_Report_Original_Submission Tuomas PuolivÃ¤li -- 7/3/2020 Reviewed

giaa106_Reviewer_1_Report_Revision_1 Tuomas PuolivÃ¤li -- 9/9/2020 Reviewed

giaa106_Reviewer_2_Report_Original_Submission Tibor Auer -- 7/13/2020 Reviewed

giaa106_Reviewer_2_Report_Revision_1 Tibor Auer -- 9/10/2020 Reviewed

giaa106_Supplemental_File
