# Supplementary material for: File-based localization of numerical perturbations in data analysis pipelines
Source: Gigascience. 2020 Dec 2;9(12):giaa106. doi: 10.1093/gigascience/giaa106 (PMC7710495; doi:10.1093/gigascience/giaa106)
Supplement: giaa106_Supplemental_File [file giaa106_supplemental_file.pdf]

# File-based localization of numerical perturbations in data analysis pipelines

Ali Salari*, Gregory Kiar, Lindsay Lewis, Alan C. Evans, Tristan Glatard

## 1. SUPPLEMENTARY MATERIAL

We used unprocessed data from 20 subjects from the HCP data releases of the 1200 Subject project available in the ConnectomeDB repository. Table S1 shows a summary of the subjects.

**Table S1. Summary of the subjects used in the experiments.**

| Subject | Release[1] | Acquisition[2] | Gender | Age |
|---|---|---|---|---|
| 101309 | S500 | Q06 | M | 26-30 |
| 102008 | S500 | Q06 | M | 22-25 |
| 102311 | S500 | Q06 | F | 26-30 |
| 103414 | Q2 | Q02 | F | 22-25 |
| 103515 | Q1 | Q02 | F | 26-30 |
| 103818 | Q1 | Q01 | F | 31-35 |
| 105014 | S500 | Q05 | F | 26-30 |
| 105115 | Q2 | Q02 | M | 31-35 |
| 106319 | Q3 | Q03 | M | 26-30 |
| 106521 | S500 | Q06 | F | 26-30 |
| 107321 | S500 | Q04 | F | 22-25 |
| 107422 | S500 | Q07 | M | 22-25 |
| 108121 | S500 | Q04 | F | 26-30 |
| 108323 | S500 | Q04 | F | 26-30 |
| 113922 | S500 | Q04 | M | 31-35 |
| 140420 | Q2 | Q02 | F | 26-30 |
| 140824 | Q3 | Q04 | M | 31-35 |
| 140925 | S500 | Q04 | F | 22-25 |
| 142424 | Q3 | Q03 | M | 26-30 |
| 142828 | Q1 | Q01 | M | 31-35 |

[1] Release refers to the HCP data release in which this subject's data was initially published to ConnectomeDB
[2] Acquisition indicates the Quarter in which this subject's data was initially acquired
