# Supplementary material for: File-based localization of numerical perturbations in data analysis pipelines
Source: Gigascience. 2020 Dec 2;9(12):giaa106. doi: 10.1093/gigascience/giaa106 (PMC7710495; doi:10.1093/gigascience/giaa106)
Supplement: giaa106_Response_to_Reviewer_Comments_Original_Submission [file giaa106_response_to_reviewer_comments_original_submission.pdf]

Author's Response To Reviewer Comments

Close

Please find the file related to the response to the reviewers and editor comments in the attached files as the 'supplementary material' under the name 'comments.docx'.

Close
