# Supplementary material for: File-based localization of numerical perturbations in data analysis pipelines
Source: Gigascience. 2020 Dec 2;9(12):giaa106. doi: 10.1093/gigascience/giaa106 (PMC7710495; doi:10.1093/gigascience/giaa106)
Supplement: giaa106_Response_to_Reviewer_Comments_Revision_1 [file giaa106_response_to_reviewer_comments_revision_1.pdf]

Author's Response To Reviewer Comments

Close

Please find the 'comments2.docx' file in the attached files in which we answered the latest comments.

Close
