# Supplementary material for: File-based localization of numerical perturbations in data analysis pipelines
Source: Gigascience. 2020 Dec 2;9(12):giaa106. doi: 10.1093/gigascience/giaa106 (PMC7710495; doi:10.1093/gigascience/giaa106)
Supplement: giaa106_Response_to_Reviewer_Comments_Revision_2 [file giaa106_response_to_reviewer_comments_revision_2.pdf]

Author's Response To Reviewer Comments

Close

Dear Editor,

We have added a section "Availability of Supporting Data" and the reference corresponding to the GigaDB DOI.
Please find the revised version of the manuscript answers to the following comments:

1. Please add a section "Availability of Supporting Data" - see PDF for details.
2. References - please update your references to include a new citation for the GigaDB DOI - it must be cited in the exact format:
Salari A; Kiar G; Lewis L; Evans AC; Glatard T: Supporting data for "File-based localization of numerical perturbations in data analysis pipelines" GigaScience Database. 2020. http://dx.doi.org/10.5524/100801

We thank you again for your time and consideration, and we remain available for any further information you may need.

Close
