# Supplementary material for: File-based localization of numerical perturbations in data analysis pipelines
Source: Gigascience. 2020 Dec 2;9(12):giaa106. doi: 10.1093/gigascience/giaa106 (PMC7710495; doi:10.1093/gigascience/giaa106)
Supplement: giaa106_Reviewer_1_Report_Original_Submission — Tuomas PuolivÃ¤li -- 7/3/2020 Reviewed [file giaa106_reviewer_1_report_original_submission.pdf]

Reviewer Report

Title: File-based localization of numerical perturbations in data analysis pipelines

Version: Original Submission Date: 7/3/2020

Reviewer name: Tuomas PuolivÃ¤li

Reviewer Comments to Author:

Ali Salari and co-authors report an interesting study about how numerical instabilities in data analysis pipelines impact reproducibility of neuroimaging experiments. The authors also provide an open-source Python-based software which can be used to detect numerical instabilities in existing pipelines. The study is overall well-conducted and the language of the manuscript is good. In addition, there does not appear to be other as systematic and practical approaches for tackling issues in numerical stability in neuroscience data analyses. Therefore, I expect this research to have broad impact. However, I also think the manuscript should be further developed, and I have below offered comments intended as constructive criticism. The present version of the manuscript also does not yet meet all requirements outlined in GigaScience author guidelines. In summary, I recommend GigaScience to publish this research upon a major revision.

Major issues:

1. The study is framed to help solving the reproducibility crisis in neuroscience but its broader background is not discussed in the current manuscript. Therefore, the reader is now left wondering about the relative importance of addressing issues in numerical stability, in comparison to other things that are known to influence reproducibility, like sample size selection, publication bias, "researcher's degrees of freedom", multiple hypothesis testing, p-hacking, and so on. I suggest the authors to discuss their research with respect to this broader context.

2. I was able to test the software using the example code available on GitHub. In that example, the software is used to compare outputs generated by grep, which is a search tool on Linux operating systems. I do understand that the authors have liked to select an easy example to introduce users to the software. However, I am now not able to comment on whether the software works in the context of the manuscript. Related to this, the participant codes of the randomly selected participants (HCP data) are not reported. I suggest the authors to provide the participant codes (e.g. as supplementary material) and an easily accessible example of how the software can be applied to a neuroimaging pipeline, ideally using a subset of the same HCP data. If all relevant details were now in a Docker container, I was not able locate them.

3. To meet the GigaScience guidelines, the methods section should be expanded by adding a section on how the software was implemented. This should include information on at least which Python version numbers are supported, and a list of libraries and their versions the work is mostly built on. For example, NumPy, SciPy, and Scikit-learn were used to develop the software but are not credited in the current manuscript. Similarly, while cited, the version of ReproZip must be reported.

4. It is reported that Dice coefficients and sizes of brain regions appear to be correlated so that numerical instabilities are focused to smaller brain structures. However, this relationship has not been

tested statistically. The authors should perform the appropriate tests.
5. In the results section, the average running times of PreFreeSurfer and FreeSurfer pipelines are not very informative unless the details of the used hardware (CPU, RAM, etc.) are also reported. I would also like to suggest reporting a measure of spread along with the average running times, output file sizes, and file access counts.
6. To meet the GigaScience statistical reporting guidelines, a rationale for using Dice coefficients must be provided. Giving the range of possible values along with descriptions of the extreme values would also help readers unfamiliar with this metric to understand the results without first resorting to other sources.
7. I am unfortunately not able to understand what is displayed in Figure 4. There are two views of a brain colored in patches of green and purple but what do the colors refer to? In the figure caption, it is stated that shown are differences between T2 fnirt results obtained using CentOS versions 6 and 7. However, what precisely are those differences? Also, please give a description in the figure caption what the two red boxes used to highlight.
8. In Figure 5, the color bar is not labeled. Also, please give a brief description of what was the variable that was binarized, so that understanding the contents of the figure is possible without referring to the main text.
9. In the introduction, it is stated that "perturbations resulting from changes in operating system versions, hardware, or parallelization parameters, may result in substantially different analysis outcomes". I would argue this to be an understatement; numerical errors are to be always expected while working with floating-point operations. To get the manuscript's points across to a wide audience, it should be emphasized that numerical stability is an issue in practically all of scientific computing. The authors could even consider referring to some example program involving a step of 'catastrophic cancellation', or the like.
10. In the abstract, it is alluded to that numerical errors are amplified in the data analysis pipelines. Is the error accumulation with respect to number of processing steps/stages linear, exponential, power-law, or something else?
11. What does the program in Listing 1 do?
Minor issues:
12. Please add a more detailed caption to Figure 6. Does each black filled circle correspond to a single participant? Reporting this would allow interpreting the figure without referring to the main text.
13. In Figure 2, what does the gray color refer to? An unsupported operation or something else?
14. In Figure 3, please write out the abbreviations or state that the same abbreviations as in Figure 2 are used.
15. Typing error in Figure 3 caption: 'ommitted'
16. The opening sentence of the introduction, 'vibrations in computational infrastructures impact data analyses' gives an impression of the manuscript being about physical movements of computing hardware influencing data analysis results. To avoid confusion, I would like to suggest rewording this sentence.
17. Are the running times or file-access counts of PreFreeSurfer and FreeSurfer correlated with the Dice coefficients?

Methods

Are the methods appropriate to the aims of the study, are they well described, and are necessary controls included? Choose an item.

Conclusions

Are the conclusions adequately supported by the data shown? Choose an item.

Reporting Standards

Does the manuscript adhere to the journal's guidelines on minimum standards of reporting? Choose an item.

Choose an item.

Statistics

Are you able to assess all statistics in the manuscript, including the appropriateness of statistical tests used? Choose an item.

Quality of Written English

Please indicate the quality of language in the manuscript: Choose an item.

Declaration of Competing Interests

Please complete a declaration of competing interests, considering the following questions:

- Have you in the past five years received reimbursements, fees, funding, or salary from an organisation that may in any way gain or lose financially from the publication of this manuscript, either now or in the future?
- Do you hold any stocks or shares in an organisation that may in any way gain or lose financially from the publication of this manuscript, either now or in the future?
- Do you hold or are you currently applying for any patents relating to the content of the manuscript?
- Have you received reimbursements, fees, funding, or salary from an organization that holds or has applied for patents relating to the content of the manuscript?
- Do you have any other financial competing interests?
- Do you have any non-financial competing interests in relation to this paper?

If you can answer no to all of the above, write 'I declare that I have no competing interests' below. If your reply is yes to any, please give details below.

I declare that I have no competing interests.

I agree to the open peer review policy of the journal. I understand that my name will be included on my report to the authors and, if the manuscript is accepted for publication, my named report including any attachments I upload will be posted on the website along with the authors' responses. I agree for my report to be made available under an Open Access Creative Commons CC-BY license (http://creativecommons.org/licenses/by/4.0/). I understand that any comments which I do not wish to be included in my named report can be included as confidential comments to the editors, which will not be published.

Choose an item.

To further support our reviewers, we have joined with Publons, where you can gain additional credit to further highlight your hard work (see: https://publons.com/journal/530/gigascience). On publication of this paper, your review will be automatically added to Publons, you can then choose whether or not to claim your Publons credit. I understand this statement.

Yes Choose an item.
