# Supplementary material for: File-based localization of numerical perturbations in data analysis pipelines
Source: Gigascience. 2020 Dec 2;9(12):giaa106. doi: 10.1093/gigascience/giaa106 (PMC7710495; doi:10.1093/gigascience/giaa106)
Supplement: giaa106_Reviewer_2_Report_Original_Submission — Tibor Auer -- 7/13/2020 Reviewed [file giaa106_reviewer_2_report_original_submission.pdf]

**Reviewer Report**

**Title: File-based localization of numerical perturbations in data analysis pipelines**

**Version: Original Submission** **Date:** 7/13/2020

**Reviewer name: Tibor Auer**

**Reviewer Comments to Author:**

General opinion
The authors address a very important yet often overlooked issue of computational instability tightly coupled with reproducibility and transparency. This issue is due to variance in implementation and software-hardware environment, which we usually take granted. Moreover, spotting the source and its effect(s) in the pipeline requires a great deal of engineering skills most researcher may not have. The authors investigated HPC pipelines partially due to their complexity. Such pipelines combining different implementations and toolboxes is becoming the norm, which renders computational instability more likely to occur and propagate, and harder to detect. The present manuscript not only raises the awareness to the computational instability, but it also provides a tool to locate it.
Major comments
The proposed solution seems to be influenced the (pre-set) order of conditions, because it takes the output Â¬files produced in the fiÂ¬rst condition as reference (in case of conflict) when testing subsequent processes. It raises the theoretical possibility of interaction across conditions, i.e. computational stability may depend on the input. Have the authors tested (e.g. by permutation) whether choosing the reference condition influences the outcome?
The findings suggest a between-subject variance in the reproducibility. However, it further implies that processes proved to be reproducible in this study may appear otherwise with different/larger datasets. In this case, only lack of reproducibility can be proved, which is a significant limitation.
Even though the proposed tool cam be considered automatic as it does not require instrumentation, its implementation and application still require certain level of engineering skills, which might deter many researchers from using it. It also requires pipelines pre-installed and pre-configured in different environment to test. The authors used containerised pipelines to circumvent the issue, which may not be an easy-to-do task for many researchers. Therefore, I am wondering what the authors suggest the target userbase for Spot would be.
According to Spot's GitHub repository (https://github.com/big-data-lab-team/spot), Boutique descriptors for the pipeline implemented in each environment to test are also required, however, it is not mentioned in the manuscript, and the format of such descriptor (or an example) is provided neither in the manuscript nor in the repo.
Minor comments
The authors stated that dataset and code are available; however, only the database is specified. I think I found the code repo at https://github.com/big-data-lab-team/HCP-reproducibility-paper, but it should be explicitly stated.
Node labels in Figure 1 are very small in the PDF. Please, make sure that they are large enough at least

for the online version of the manuscript.
How was "importance" defined for findings showed in Figure 4?

**Methods**

Are the methods appropriate to the aims of the study, are they well described, and are necessary controls included? Choose an item.

**Conclusions**

Are the conclusions adequately supported by the data shown? Choose an item.

**Reporting Standards**

Does the manuscript adhere to the journal's guidelines on minimum standards of reporting? Choose an item.

Choose an item.

**Statistics**

Are you able to assess all statistics in the manuscript, including the appropriateness of statistical tests used? Choose an item.

**Quality of Written English**

Please indicate the quality of language in the manuscript: Choose an item.

**Declaration of Competing Interests**

Please complete a declaration of competing interests, considering the following questions:

- Have you in the past five years received reimbursements, fees, funding, or salary from an organisation that may in any way gain or lose financially from the publication of this manuscript, either now or in the future?
- Do you hold any stocks or shares in an organisation that may in any way gain or lose financially from the publication of this manuscript, either now or in the future?
- Do you hold or are you currently applying for any patents relating to the content of the manuscript?
- Have you received reimbursements, fees, funding, or salary from an organization that holds or has applied for patents relating to the content of the manuscript?
- Do you have any other financial competing interests?
- Do you have any non-financial competing interests in relation to this paper?

If you can answer no to all of the above, write 'I declare that I have no competing interests' below. If your reply is yes to any, please give details below.

I have co-authored several papers with TG. TG and I are members of the INCF NIDASH. TG is the Treasurer Elect of the OHBM Open Science Special Interest Group. This group organised the ORS2020 I also volunteered to. Also I am currently applying to the Chair Elect  role of this group.

I agree to the open peer review policy of the journal. I understand that my name will be included on my report to the authors and, if the manuscript is accepted for publication, my named report including any attachments I upload will be posted on the website along with the authors' responses. I agree for my report to be made available under an Open Access Creative Commons CC-BY license (http://creativecommons.org/licenses/by/4.0/). I understand that any comments which I do not wish to be included in my named report can be included as confidential comments to the editors, which will not be published.

Choose an item.

To further support our reviewers, we have joined with Publons, where you can gain additional credit to further highlight your hard work (see: https://publons.com/journal/530/gigascience). On publication of this paper, your review will be automatically added to Publons, you can then choose whether or not to claim your Publons credit. I understand this statement.

Yes Choose an item.
