# Supplementary material for: File-based localization of numerical perturbations in data analysis pipelines
Source: Gigascience. 2020 Dec 2;9(12):giaa106. doi: 10.1093/gigascience/giaa106 (PMC7710495; doi:10.1093/gigascience/giaa106)
Supplement: giaa106_Reviewer_2_Report_Revision_1 — Tibor Auer -- 9/10/2020 Reviewed [file giaa106_reviewer_2_report_revision_1.pdf]

Reviewer Report

Title: File-based localization of numerical perturbations in data analysis pipelines

Version: Revision 1 **Date:** 9/10/2020

Reviewer name: Tibor Auer

Reviewer Comments to Author:

The authors have considered all my comments and answered them in great details. The corresponding changes have greatly improved the manuscript.
I have only one minor comment which I do not find absolutely necessary to amend before publication. I leave the decision to the editor:
- As the authors admit in their response, the target users of the Spot tool are not the end-users and the researchers themselves but rather the pipeline and platform developers who are likely to have the skills necessary to e.g. build Docker containers. I would prefer this note mentioned in the manuscript to 1) better target the audience and 2) avoid potential frustration in users who might start using the tool without expecting difficulty.

Methods

Are the methods appropriate to the aims of the study, are they well described, and are necessary controls included? Choose an item.

Conclusions

Are the conclusions adequately supported by the data shown? Choose an item.

Reporting Standards

Does the manuscript adhere to the journal's guidelines on minimum standards of reporting? Choose an item.

Choose an item.

Statistics

Are you able to assess all statistics in the manuscript, including the appropriateness of statistical tests used? Choose an item.

Quality of Written English

Please indicate the quality of language in the manuscript: Choose an item.

Declaration of Competing Interests

Please complete a declaration of competing interests, considering the following questions:

- Have you in the past five years received reimbursements, fees, funding, or salary from an organisation that may in any way gain or lose financially from the publication of this manuscript, either now or in the future?
- Do you hold any stocks or shares in an organisation that may in any way gain or lose financially from the publication of this manuscript, either now or in the future?
- Do you hold or are you currently applying for any patents relating to the content of the manuscript?
- Have you received reimbursements, fees, funding, or salary from an organization that holds or has applied for patents relating to the content of the manuscript?
- Do you have any other financial competing interests?
- Do you have any non-financial competing interests in relation to this paper?

If you can answer no to all of the above, write 'I declare that I have no competing interests' below. If your reply is yes to any, please give details below.

I have co-authored papers with TG. TG and I are members of the INCF NIDASH Task Force (http://nidm.nidash.org). TG is the Treasurer Elect of the OHBM OSSIG. This group organised the ORS2020 I also volunteered to. Also I am currently applying to the Chair Elect role of this group.

I agree to the open peer review policy of the journal. I understand that my name will be included on my report to the authors and, if the manuscript is accepted for publication, my named report including any attachments I upload will be posted on the website along with the authors' responses. I agree for my report to be made available under an Open Access Creative Commons CC-BY license (http://creativecommons.org/licenses/by/4.0/). I understand that any comments which I do not wish to be included in my named report can be included as confidential comments to the editors, which will not be published.

Choose an item.

To further support our reviewers, we have joined with Publons, where you can gain additional credit to further highlight your hard work (see: https://publons.com/journal/530/gigascience). On publication of this paper, your review will be automatically added to Publons, you can then choose whether or not to claim your Publons credit. I understand this statement.

Yes Choose an item.
